# Shifting breast cancer surveillance from current hospital setting to a community based setting: a cost-effectiveness study

**DOI:** 10.1186/s12885-018-3992-7

**Published:** 2018-01-24

**Authors:** Kelly M. de Ligt, Annemieke Witteveen, Sabine Siesling, Lotte M. G. Steuten

**Affiliations:** 1 Dept. of Research, Netherlands Comprehensive Cancer Organisation (IKNL), Godebaldkwartier 419, 3511 DT Utrecht, the Netherlands; 2 Dept. of Health Technology and Services Research, MIRA Institute for Biomedical Technology and Techical Medicine, University of Twente, Drienerlolaan 5, 7522 NB Enschede, the Netherlands; 3 Fred Hutchinson Cancer Research Center, HICOR: Hutchinson Institute for Cancer Outcomes Research, 1100 Fairview Ave. N, Seattle, WA 98109 USA

**Keywords:** Breast cancer, Cost-effectiveness, Loco regional recurrence, Surveillance, Screening

## Abstract

**Background:**

This study explores the effectiveness and cost-effectiveness of surveillance after breast cancer treatment provided in a hospital-setting versus surveillance embedded in the community-based National Breast Cancer Screening Program (NBCSP).

**Methods:**

Using a decision tree, strategies were compared on effectiveness and costs from a healthcare perspective over a 5-year time horizon. Women aged 50–75 without distant metastases that underwent breast conserving surgery in 2003–2006 were selected from the Netherlands Cancer Registry (*n* = 14,093). Key input parameters were mammography sensitivity and specificity, risk of loco regional recurrence (LRR), and direct healthcare costs. Primary outcome measure was the proportion true test results (TTR), expressed as the positive and negative predictive value (PPV, NPV). The incremental cost-effectiveness ratio (ICER) is defined as incremental costs per TTR forgone.

**Results:**

For the NBCSP-strategy, 13,534 TTR (8 positive; 13,526 negative), and 12,923 TTR (387 positive; 12,536 negative) were found for low and high risks respectively. For the hospital-based strategy, 26,663 TTR (13 positive; 26,650 negative) and 24,883 TTR (440 positive; 24,443 negative) were found for low and high risks respectively. For low risks, the PPV and NPV for the NBCSP-based strategy were 3.31% and 99.88%, and 2.74% and 99.95% for the hospital strategy respectively. For high risks, the PPV and NPV for the NBCSP-based strategy were 64.10% and 98.87%, and 50.98% and 99.71% for the hospital-based strategy respectively. Total expected costs of the NBCSP-based strategy were lower than for the hospital-based strategy (low risk: €1,271,666 NBCSP vs €2,698,302 hospital; high risk: €6,939,813 NBCSP vs €7,450,150 hospital), rendering ICERs that indicate cost savings of €109 (95%CI €95–€127) (low risk) and €43 (95%CI €39–€56) (high risk) per TTR forgone.

**Conclusion:**

Despite expected cost-savings of over 50% in the NBCSP-based strategy, it is nearly 50% lower accurate than the hospital-based strategy, compromising the goal of early detection of LRR to an extent that is unlikely to be acceptable.

## Background

Breast cancer is the most common type of cancer among women globally [1] and in the Netherlands [2] and the most common cancer site among female cancer survivors [3]. As both incidence and survival have increased over the last decade, prevalence is rising [2]. One important contributor to the reduction in breast cancer mortality is patient surveillance for early detection of loco-regional recurrences (LRR) and second primary (SP) tumours [2, 4]. In the Dutch national guideline on breast cancer (NABON guideline) surveillance schemes consist of physical examination and annual mammography and take place in the hospital for five years after treatment [5]. These schemes are comparable to surveillance schemes in other countries, such as the United Kingdom [6], Australia [7] and the United States [8].

With a growing prevalent population requiring surveillance, the question how to allocate the required resources such that the surveillance health benefits are maximised, becomes more pertinent [9, 10]. Debate is ongoing about the frequency and duration of surveillance, and what the most appropriate care provider is to perform surveillance. Also, the most effective way to detect recurrences or SP tumours has not been firmly established. While women feel reassured by attending the breast cancer clinic [11], within a hospital setting no clinical benefits have been demonstrated for high-intensity, longer duration or high-frequency surveillance schemes compared to schemes with lower resource demands. Studies also showed that surveillance can effectively be provided outside the hospital by general practitioners or nurses, and could for example be incorporated in a national screening program [12–15]. Notably, Lu et al. [16] concluded that the detection rate of small tumours in a community-based surveillance strategy was comparable to a hospital-based strategy. In the National Breast Cancer Screening Programme (NBCSP), established in 1990, healthy women age 50–75 are screened biennially for early-stage breast cancer. Screening is done by mammography and takes place in mobile screening busses that call on communities across the country [5, 17].

The aim of our study is to explore the effectiveness and expected cost-effectiveness of current standard hospital-based breast cancer surveillance versus breast cancer surveillance embedded in the community-based screening program after one year of common hospital-based surveillance, over a time-horizon of five years post-treatment.

## Methods

Study population: Patients were selected from the Netherlands Cancer Registry (NCR), a nationwide population-based registry which records all newly diagnosed tumours since 1989. The database collects extensive information on primary tumours and recurrences, and is representative for the Dutch population. Women age 50 to 75 (which are the NBCSP age criteria for participation) diagnosed with breast cancer between 2003 and 2006 and treated with Breast Conserving Surgery (BCS) were selected; women treated without curative intent (no surgery or with macroscopic residual disease after surgery), with distant metastases, previous or synchronous tumours (diagnosed within three months after the first tumour), or treated with neo-adjuvant systemic therapy were excluded. Adjuvant treatment should have been applied in case of microscopic residue; for patients that underwent neo-adjuvant treatment, risk could not be calculated (also see the ‘measurement of effectiveness’-section). In the final analysis, 14,093 patients were included.

Analytic perspective, time horizon, and comparators: Both strategies, as described below, are compared on effects and costs using a healthcare perspective, since the majority of costs are captured in this perspective. A five year time horizon was applied as most recurrences are known to occur within five years after primary treatment [8, 18]; besides, the current guideline recommends surveillance for a time period of five years to be safe and effective [5]. Both strategies are compared from the second year on: during the first year the surveillance not only aims for cancer detection, but also addresses potential post-treatment complications of physical and psychological nature [5], which can only be provided at a hospital and not at the NBCSP, and is therefore not taken into account in this study. From year two onwards, the surveillance is either hospital or community-based. In line with the Dutch pharmaco-economic guidelines, future costs and effects were annually discounted at 4% and 1.5% respectively, since this article focuses specifically surveillance and screening as provided in the Netherlands.

Both the current standard hospital-based surveillance strategy and the hypothetical NBCSP-based surveillance strategy consist of frequent mammographic imaging; four mammograms are taken per appointment (cranial caudal and medio lateral oblique on both breasts). All women with a positive mammogram, either taken at the hospital or the NBCSP, are referred to the hospital to get an additional diagnostic consultation including ultrasound and puncture [17]. The strategies differ in use of resources. In the hospital mammograms are taken and interpreted by a radiologist, mammograms in the NBCSP-embedded surveillance are taken by specially trained nurses and assessed by two independent radiologists. Also, hospital-embedded surveillance takes place at the hospital, whereas NBCSP-based surveillance is carried out at NBCSP-screening busses. Additionally, there is a difference in surveillance frequency: hospital surveillance takes place annually, while NBCSP-based surveillance takes place biennially. It is assumed that the NBCSP-based surveillance will be incorporated in the current screening schedules of the NBCSP with busses available across the country. Although NBSCP is financed by the government as preventive measure, surveillance in the NBCSP-based setting for breast cancer patients is covered by all health insurance companies.

Choice of health outcomes: The primary effectiveness measure was the proportion of true test results (TTR), expressed as the positive predictive value (PPV) and the negative predictive value (NPV). The secondary outcome measure was the total number of true positive and true negative test results. The ICER is defined as incremental costs (difference in costs of two interventions) per TTR forgone (difference in effects of two interventions) [19].

### Measurement of effectiveness

Sensitivity and specificity of 0.654 and 0.983 respectively were applied for both hospital-based strategy and NBCSP-based strategy (Table 1). These input data were based on Houssami et al. [20], in which the performance of screening mammography was tested in both women with and without a personal history of early-stage breast cancer. Double-reading by NBCSP-assessors in the NBCSP-based strategy increases the sensitivity [21], but the sensitivity is lowered by their higher reading speed; therefore the assumption was made that sensitivity and specificity were comparable for hospital-based strategy and NBCSP-based strategy.

**Table 1:** Model parameters

Parameter	Input	Source
Risk on LRR, year 2–5		Witteveen et al. [18]
• Low risk	Yr 2: 0.0003, Yr 3: 0.0006, Yr 4: 0.0002, Yr 5: 0.0011	
• High risk	Yr 2: 0.0204, Yr 3: 0.0345, Yr 4: 0.0076, Yr 5: 0.0086	
Sensitivity	0.654 (95%CI: 0.61–0.69)	Houssami et al. [20]
Specificity	0.983 (95%CI: 0.982–0.984)	
Costs per unit (€)		
Cost of examination in NSP (mammography)	64.00 (25% range: €48 - €80)	RIVM [17]
Cost of examination in hospital (mammography)	82.89 (25% range: €62 - €104)	Calculation from publicly accessible hospital price lists for 2013.
Cost of add. examination in hospital after false positive result	926.83 (25% range: €695 - €1185)	
Treatment costs for early detected LRR	9705 (25% range: €7280 – €12,122)	
Treatment costs for late detected LRR	15,515 (25% range: €11,640 – €19.,384)	

Breast cancer recurrences are classified as local recurrences (LR), regional recurrences (RR), SP tumours or distant metastasis (DM). Presence of a LR (any epithelial breast cancer in the ipsilateral breast) and/or RR (any breast cancer in the ipsilateral lymph nodes) is defined as a LRR [22]. Only first or synchronous LRRs were included in this study. Information on the population was retrieved from the NCR. Based on literature and availability of data within the NCR, potential risk factors were selected. The final selection of risk factors was found by use of backward elimination. Risk of LRR per year was calculated in STATA 13 by multivariable logistic regression. Tests were performed to check for interaction and correlation as previously described in more detail by Witteveen et al. [18].

In the model, we assumed that the entire population (*n* = 14,093) was either low risk or high risk. We decided to simulate these two extremes, in order to estimate the possible range of outcomes for both strategies. Outcomes for both risk groups were compared. Low and high risk were calculated by using the three most influencing risk factors for patients aged 50–75, since women in this age bracket are initially invited for the screening programme. The low risks group consisted of women with grade 1 tumours, no node involvement, and hormonal treatment. The high risk group consisted of women with grade 3 tumours, over three nodes involved, and without hormonal treatment. Low and high risk per group are shown in Table 1. All cause and breast cancer mortality were low and the same in both strategies and therefore not taken into account [1, 23, 24].

### Estimating resources and costs

Resource use for hospital-based strategy was derived from the Dutch national guideline on breast cancer [5] and expert opinion. Resource use for the NBCSP-based surveillance was also based on the guideline, the official website of the screening programme [17], and a site visit plus interviews at a mobile screening unit. Average costs of each activity at the hospital were calculated from costs from publicly accessible hospital price lists (*n* = 12) from several hospitals. Costs of hospital-based mammography were €83 per woman. Costs of a single screening visit were estimated at €64 per woman and were retrieved from the official website of the screening programme [17], calculated by dividing total costs of the programme by the number of women that were screened within a year. When women were tested false positive, extra diagnostic tests were used unnecessarily, which was estimated at €927 per false positive tested woman (consisting of an ultrasound, puncture and consult, addressed in this article as costs for extra diagnostic tests).

Treatment costs for early and late detected LRR were calculated from publicly accessible hospital price lists, and were estimated at €9705 and €15,515 respectively (early: intensive radiotherapy €9705; late: mastectomy and intensive radiotherapy €9705 + €5809 = €15,515). The guideline states that treatment of recurrences is dependent on the characteristics of the recurrence. A LRR was defined as an early detected LRR when it was detected during the year it developed; a LRR was defined as a late detected LRR when it was detected after this year. Since it was impossible to calculate costs for all subgroups of women within the early and late detected women, it was assumed that all women receive the same type of treatment for early detected LRR, and the same treatment for late detected LRR. All costs were in 2013 euros and are presented in Table 1.

**Fig. 1:**
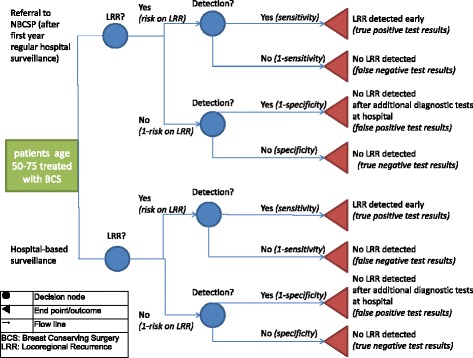
Decision tree NBCSP-based surveillance vs hospital-based surveillance

Choice of model and key assumptions: A decision tree was developed in Microsoft Excel 2010 to compare the expected effects and costs of the current hospital-based surveillance strategy to NBCSP community-based surveillance strategy (Fig. 1). We assumed 100% compliance to the surveillance programmes in both strategies. Further, it was assumed that mammograms were exchanged between the hospital and the NBCSP (in case of referral from one setting to another), and hospital and NBCSP mammography were comparable in performance. A LRR could only be detected by mammography during appointments, or not at all, which means that the possibility of interval LRRs was not included in the model. If LRRs were missed during screening, 100% of them were assumed to be detected the next screening round, since LRRs continue to grow and so do its chances of detection.

### Analytical methods

The PPV was calculated by dividing the number of positive TTR by the total number of positive test results. The NPV was calculated by dividing the number of negative TTR by the total number of negative test results. The ICER was calculated by dividing the difference in costs by the difference in the number of TTR between both strategies. Fieller’s Theorem was used to determine the 95% confidence interval around the ICER.

One-way sensitivity analyses were carried out on the following parameters: hospital mammography sensitivity and specificity, NBCSP mammography sensitivity and specificity, costs of hospital mammography and costs of NBCSP mammography. The range of diagnostic parameter estimates was based on published variance estimates; standard ranges of 25% above and below median cost estimated was assumed [19].

## Results

**Fig. 2:**
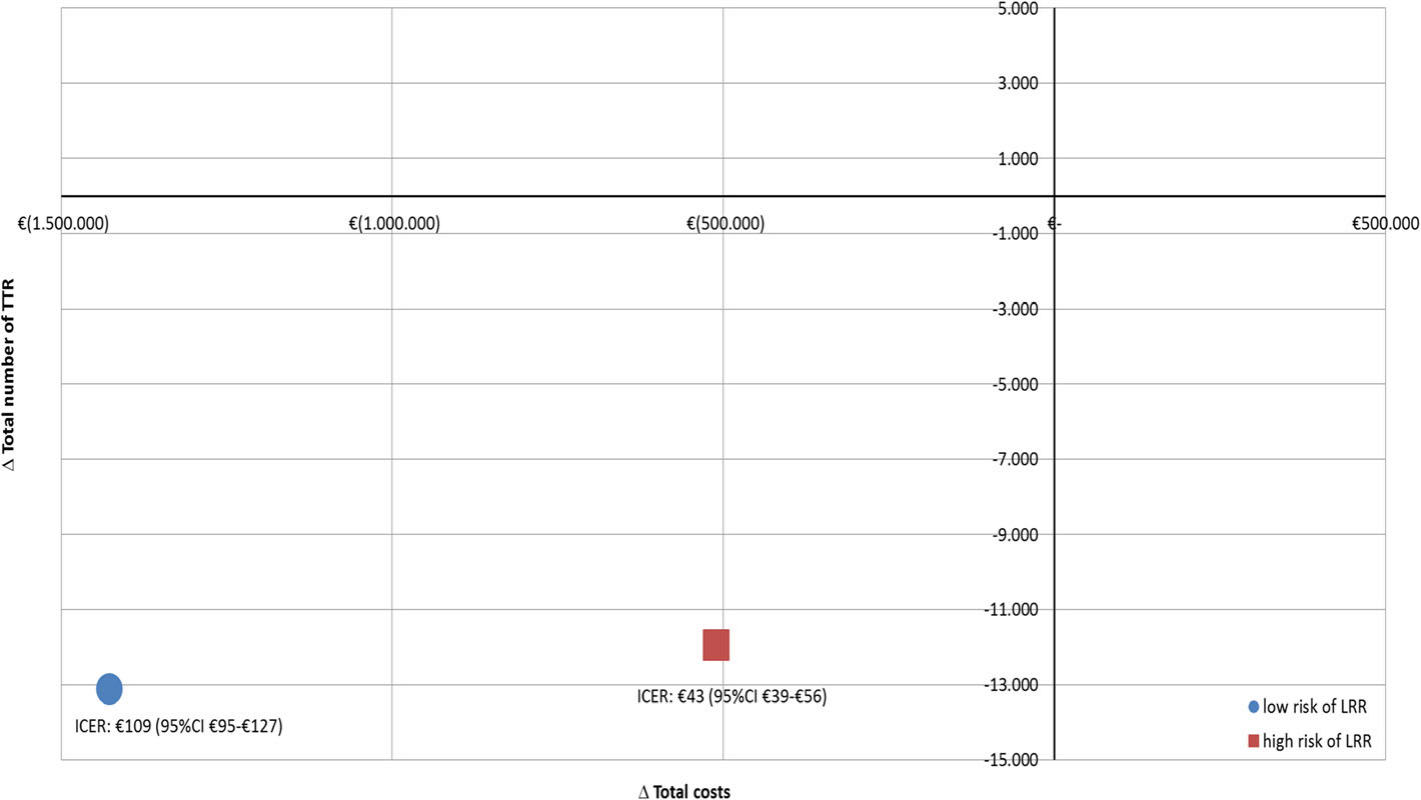
ICER-plane for low and high risk of LRR (Δ total costs, Δ total TTR)

Discounted results for the base case model are presented in Table 2 and Fig. 2. For low risks, the PPV and NPV for the NBCSP-based strategy were 3.31% and 99.88%, and 2.74% and 99.95% for the hospital strategy respectively. For high risks, the PPV and NPV for the NBCSP-based strategy were 64.10% and 98.87%, and 50.98% and 99.71% for the hospital-based strategy respectively. In the NBCSP-based strategy, 8 positive TTRs and 13,526 negative TTRs were found for low risk, and 387 positive TTRs and 12,536 negative TTRs for high risk patients. For the hospital-based strategy, 13 positive TTRs and 26,650 negative TTRs and 440 positive TTRs and 24,443 negative TTRs were found for low and high risk patients respectively.

**Table 2:** Results base case model per surveillance strategy for low and high risk of LRR

	Hospital-based strategy				NBCSP-based strategy			
	Low risk of LRR		High risk of LRR		Low risk of LRR		High risk of LRR	
Total number of TTR (positive TTR, negative TTR)	26,663		24,883		13,534		12,923	
	13 (0.2%)	26,650 (99.8%)	440 (6.5%)	24,443 (93.5%)	8 (0.1%)	13,526 (99.9%)	387 (5.7%)	12,536 (94,3%)
Total costs (€) surveillance	2,529,150		2,367,616		1,063,530		1,013,224	
Total costs (€) of treatment	169,152		5,082,534		208,136		5,926,590	
Final costs (€)	2,698,302		7,450,150		1,271,666		6,939,814	
Early vs late detection of LRR (%)	10 early, 3 late (0.14, 0.04)		298 early, 142 late (4.39, 2.09)		2 early, 6 late (0.03, 0.09)		113 early, 274 late (1.66, 4.03)	
Self-detected LRRs after 5 years (%)	3 (0.04)		17 (0.25)		8 (0.11)		66 (0.97)	
False positive test results resulting in extra diagnostic tests (%)	461 (6.55)		423 (6.23)		234 (3.32)		217 (3.19)	

TTR = True (positive and/or negative) Test Results

LRR = Locoregional Recurrence

Total costs of €1,271,666 for NBCSP-based strategy and €2,698,302 for hospital-based strategy were found for low risk patients, and total costs of €6,939,813 for NBCSP-based strategy and €7,450,150 for hospital-based strategy were found for high risk patients. From this follows an ICER of €109 (95%CI €95–€127) saved per TTR forgone for low risk patients (13,534 TTR for NBCSP, 26,663 TTR for hospital), and an ICER of €43 (95%CI €39–€56) saved per TTR forgone for high risk patients (12,923 TTR for NBCSP, 24,883 TTR for hospital). The cost-effectiveness plane (Fig. 2) shows the difference in total costs on the X-axis and the difference in TTR on the Y-axis between the strategies, stratified by low and high risk of LRR, providing a visual presentation of the ICERs.

Total costs consisted of surveillance costs and treatment costs. Costs for low-risk patients attaining hospital-based surveillance and treatment were €2,529,150 and €169,152 respectively; costs for NBCSP-based surveillance and treatment were €1,063,530 and €208,136 respectively. Costs for high-risk patients attaining hospital-based surveillance and treatment were €2,367,616 and €5,082,534 respectively; costs for NBCSP-based surveillance and treatment were €1,013,223 and € 5,926,590 respectively.

This difference in surveillance programme costs is mainly the result of the lowered frequency of the NBCSP-based strategy; the difference in treatment costs is caused by the increased amount of late and self-detected recurrences in the NBCSP-based strategy, as a result of this lowered surveillance frequency. Compared to hospital-based strategy, NBCSP-based strategy led to almost twice as much late detected LRRs (3 vs 6 and 142 vs 274 for low and high risk patients respectively); the number of detected LRRs after terminating surveillance was three (3 vs 8) and four (17 vs 66) times higher for low and high risk respectively. Only half as much women received extra diagnostic tests compared to hospital strategy (low risk: 234 vs 461, high risk: 217 vs 423), thus decreasing the costs for the NBCSP-based strategy.

**Fig. 3:**
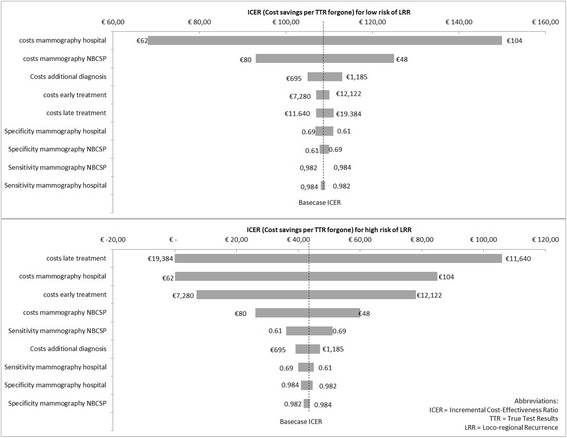
ICER tornado diagram for low risk (above) and high risk (below) of LRR

A one-way sensitivity analysis (Fig. 3) showed that for the low risk group the model outcomes are most sensitive to the costs of mammography in both the NBCSP-based as hospital-based setting: the lower bound specificity input for hospital based mammography costs (0.982) results in an ICER of €62 saved per TTR forgone and the higher bound input of (0.984) in an ICER indicating €104 saved per TTR forgone. In the NBCSP-based strategy the sensitivity analysis indicated NBCSP to be inferior (i.e. less effective) at the lower bound input value for mammography specificity costs (0.982) saving €80 per TTR forgone and at it’s higher bound input (0.984) saving €48 per TTR forgone. In the high risk group, costs of late and early treatment and the costs of mammography have a high impact on the model outcomes, contrary to the low risk group. None of the sensitivity analyses, however, indicate a different recommendation than the one arrived at in the base case analysis.

## Discussion

This model-based analysis compared the effectiveness and cost-effectiveness of an NBCSP-embedded surveillance strategy to the current hospital-based surveillance for breast cancer patients. Since the five-year risk on LRR decreased over the last decades, a less frequent surveillance strategy was expected to be suitable. The NBCSP-based strategy was expected to be cost-saving, since costs were halved, but the also accuracy was expected to be reduced, since the number of TTR was halved as well. Notably, the analysis showed that LRRs would more often be self- or late-detected for NBCSP-based surveillance, which could possibly influence survival. The cost-effectiveness trade-off therefore is one of “willingness to accept”, instead of “willingness to pay”. Specifically: is society willing to accept less accuracy for a large reduction of costs? To address this question, it is necessary to consider the potential implications of lower accuracy for patient survival and Health-Related Quality of Life (HRQoL). As Health Related Quality of Life estimates for early and late detection of recurrences were not available, the ICERs as calculated in this study reflect the difference in total costs and the difference in number of TTR, not Quality Adjusted Life Years (QALYs). Hence, an agreed upon range of willingness-to-pay or willingness-to-accept threshold is not available. Women with recurrences have a lower HRQoL [25], and it is to be expected that early detected and treated recurrences are associated with higher QALYs than late detected and treated recurrences. Since the NBCSP-based strategy was considered less effective, adding HRQoL-estimates to our model is not likely to change the conclusion on preferring hospital-based surveillance over NBCSP-based surveillance, and would therefore not provide additional information to our simplified model. Patients’ preferences for each surveillance strategy are important to assess as well, but are more complex to predict. As women have a preference for follow-up provided by a specialist [26], women may appreciate the hospital-based strategy more than the quick surveillance process of the NBCSP-based strategy. On the other hand, the lower surveillance frequency of the NBCSP means they are less often confronted with their disease, and women with lower risks accept less visits when the risk is effectively communicated [27]. Research into preferences for surveillance is needed to inform QALY calculations and the discussion whether less accuracy is acceptable. Furthermore, we chose a healthcare perspective, thus not including travel time and costs. With almost every hospital in the Netherlands providing breast cancer care, there is a high geographical density in surveillance locations. Therefore, we expect travel time and costs not to decrease drastically in the NBCSP-based strategy compared to the hospital setting and considered this therefore less relevant. Furthermore, as the Netherlands has a predominantly private health insurance market that is mandated by the government to cover a basic package of healthcare services to all citizens, which includes breast cancer screening and surveillance regardless of the setting in which this is provided, there would be no difference in access or coverage for individual patients based on their specific health insurance plan.

Several other publications discussed less intensive surveillance after breast cancer, and found equal survival outcomes [11–13, 15]. Most of the articles included in the review of surveillance care by Collins et al. [12], as well as the study from Smith et al. [13] compared more intensive surveillance to the standard surveillance, which resulted in favour of the standard, less intensive surveillance. More similar to our study, Lu et al. [16] simulated a population of breast cancer patients to evaluate less intensive surveillance strategies, amongst others by earlier referral to a NBCSP. They conclude this does not lead to a decrease in the detection of small tumours. Besides only looking at SP tumours, Lu et al. did not take into account the negative TTRs. Our study found that although an NBCSP-based strategy led to a comparable amount of true positive test results, more LRR were late or self-detected, which could impact survival. The NBCSP-based strategy was also less accurate than the hospital-based strategy, since negative TTRs were halved. Studies looking at other surveillance strategies, as for example GP-led surveillance, found similar effectiveness, but did not consider different intensities of surveillance [14–16]. It has to be noted that surveillance in some other countries than the Netherlands are more intensive. For example, in the United States patients are in general seen every three to four months up to three years after treatment, and once or twice per year after that [8]. Conclusions that surveillance can safely be de-intensified have to be considered in light of the baseline level of surveillance intensity in that setting.

This study has a number of strengths worth mentioning. First, it considers the large heterogeneity in breast cancer survivors undergoing surveillance by stratifying the analysis for high and a low risk group, to assess the effect and potential differences between those extremes. For both risk groups, the results suggest that a shift of surveillance to the NBCSP-setting is not the preferable option. While the lower accuracy in the NBCSP-setting would lead to less serious consequences in the low risk versus the high risk group, the recommendation against using a NBCSP-strategy holds for both groups. That said, although the lower intensity NBCSP-setting does not provide a good alternative for surveillance in low risk groups, other less intensive and personalized options should still be explored. To move towards more personalized health care in practice, information on cost effectiveness and viability is necessary [28], and this study contributes to that. Second, a very large population retrieved from the NCR was modelled, meaning that the generalizability of the study findings to the real world population of the Netherlands is high and specific to low and high risk subgroups.

Some of the assumptions made, need further discussion here. First, we assumed that hospital-based and NBCSP-based mammography were comparable in performance. The sensitivity analyses show that while mammography specificity inputs are influential on the model outcomes, the conclusion regarding NBCSP being the less effective option remains under all plausible inputs and it may even be dominated by the hospital-based strategy. We chose our input based on the article of Houssami et al. [20], since sensitivity is lower for patients with a history of breast cancer compared to a healthy screening population. Although this study was based in the United States, where breast cancer screening and surveillance are organised differently than in The Netherlands, we considered these data to best fit our model objectives. Furthermore, it is important to state that the analysis considers an surveillance strategy embedded in the existing NBCSP and its results cannot be generalized to potential future adaptations of the NBCSP for all or specific subgroups of women.

Second, this study assumed 100% compliance, which is unlikely in real practice. Ghezzi et al. [29] found a compliance of more than 80% for both an intensive and less intensive surveillance protocol. At a median surveillance of 71 months, no difference was apparent in overall survival with 132 deaths (20%) in the intensive group and 122 deaths (18%) in the control group. We have no data that suggest that non-compliance rates would differ between both strategies. If NBCSP-based surveillance would lead to less compliance than for hospital-based strategy, the relative effectiveness of the hospital-based strategy further increases, strengthening our conclusion. If the compliance would be higher for NBCSP-based strategy, the effectiveness would increase in a degree too small to outperform hospital-based surveillance, leaving the conclusion unaltered.

Also, detecting recurrences in between surveillance appointments was not modelled in this study, which led to overestimation of the performance of both strategies. Approximately 40% of recurrences is detected during routine visits or routine tests in asymptomatic patients [30]. Since NBCSP-based surveillance consists of biannual visits, the percentage of interval-detected recurrences is expected to be even higher, overestimating the performance of the NBCSP-based strategy more than the hospital-based strategy. This would make the NBCSP-strategy even less preferable then already concluded.

Since patients without recurrence should have the same survival irrespective of surveillance strategy, we did not include breast cancer specific or overall mortality. Breast cancer-related mortality is decreasing in many countries because of earlier diagnosis and improved treatment modalities [1, 23]; all-cause mortality in our input population was about 12%. Doyle et al. [24] found no difference in cause-specific and overall survival after a recurrence in the first five years and only a 3% difference after ten years. In case of a recurrence, it is expected that survival will differ between the strategies, as recurrences are on average detected at a later stage in the NBCSP-strategy. If we would have included this difference in survival, based on greater effectiveness the preference for hospital-based surveillance would even be higher.

As a final remark, we would like to emphasize that this study, as all model-based analyses, does not capture the full complexity of real-world practice; hence assumptions were inevitable to reflect the most salient aspects of an alternative surveillance arrangement that are reflective of the decision problem. Although we have compared two health care services that execute similar imaging activities, it should be kept in mind that both services have a rather contradicting goal. The analysis compares annual surveillance provided in a hospital-setting versus biannual surveillance embedded in a community-based screening programme. The latter is set up for a specific purpose (population screening) and designed, in terms of screening intervals (as well as such features as threshold values), as an efficient means of achieving its original purpose, not the proposed new one. Therefore, comparision of both stategies would ideally include more indicators than only incremental costs and the number of TTR. Chosen indicators might not reflect strenghts and limitations of both services in an equal way. Ideally, a surveillance service has a low rate of false positive test results, which is not achievable for a screening service, since that would mean a lower detection rate. Besides, although it is understood that false positives are an inevitable effect of a high detection rate, we decided to assign costs to every false positive event: in practise, these costs are made as well.

While the reported estimates of incremental costs and effects result from a health economic analysis that has been performed in accordance with broadly accepted health economic guidelines, interpretation and translation of these findings to the variety and complexity of real world screening and surveillance contexts, requires caution. We postulate this study as an incentive for further debate and research regarding personalized and cost-effective strategies for cancer surveillance.”

## Conclusion

The NBCSP-based surveillance strategy cuts costs in half but also the number of TTRs, compared to a hospital-based surveillance strategy. The ICERs indicate cost savings of €109 (95%CI €95–€127) and €43 (95%CI €39–€56) per TTR forgone for low and high risk patients respectively. Further, the NBCSP-based strategy led to twice as much late detected LRRs, three to four times more self-detected LRRs after termination of surveillance, and a reduction in diagnostic tests. While a NBCSP-based strategy could lower direct health care costs, it goes against the goal of early detection of LRRs and improving outcomes, since it leads to only half of the true test results compared to hospital-based strategy and an increase in late and self-detected LRR.

## Abbreviations

BCS: Breast Conserving Surgery
DM: Distant Metastasis
HRQoL: Health Related Quality of Life
ICER: Incremental Cost-Effectiveness Ratio
LR: Local Recurrence
LRR: Loco-Regional Recurrence
NBCSP: National Breast Cancer Screening Program
NCR: National Cancer Registry
NPV: Negative Predictive Value
PPV: Positive Predictive Value
QALY: Quality Adjusted Life Years
RR: Regional Recurrence
SP: Second Primary
TTR: True Test Result
